# Country-level determinants of healthcare access for refugee populations in a host country: a comparative case study

**DOI:** 10.1186/s12939-026-02915-x

**Published:** 2026-06-06

**Authors:** Sarah Yeo, SeYeon Kim, Gohtbyeol Kim, Pratibha Bhandari, Angela Dawson

**Affiliations:** 1 The University of Arizona Cancer Center, 1515 N. Campbell Ave, Tucson, AZ USA; 2 School of Public Health, Seoul National University, Seoul, South Korea; 3 School of Nursing and Midwifery, Faculty of Health, University of Technology Sydney, Sydney, Australia; 4 School of Public Health, Faculty of Health, University of Technology Sydney, Sydney, Australia

**Keywords:** Refugee health, Host country, United States, South Korea, Australia, Refugee policy, Case study

## Abstract

**Background:**

Given the unprecedented global volume of forced migration, ensuring equitable access to healthcare for refugees has become a pressing global health priority. This comparative case study examined country-level determinants that influence healthcare access and social determinants of health among refugees in Australia, South Korea, and the United States.

**Methods:**

We conducted a comparative case study to identify country-level factors influencing refugee healthcare access in Australia, South Korea, and the United States. Countries were selected to capture diversity in geographic context, immigration and integration policies, and healthcare system structures. Data sources included a comprehensive review of laws, policies, and peer-reviewed literature, as well as in-depth interviews with refugees, healthcare providers, resettlement agency staff, and legal professionals. We used framework analysis guided by a conceptual model, incorporating both deductive and inductive coding. This study focused on cross-case themes emerging from the synthesis of all data sources.

**Results:**

Findings revealed that, while refugees in all three host countries generally view health services as superior to those in their countries of origin, significant disparities persisted in access across various factors. The availability of culturally and linguistically appropriate care, legal frameworks, insurance systems, and social supports varied widely, contributing to health disparities. In the United States, complex healthcare navigation and insurance gaps posed substantial barriers, while in South Korea, legal mandates for interpretation services were lacking, and health insurance coverage was limited for certain refugee groups. Australia’s universal health coverage and government-funded language and interpretation services supported more inclusive access, although barriers existed for vulnerable subgroups.

**Conclusions:**

This study underscores the significance of national policies, culturally competent care, and long-term integration support in promoting health equity for refugees. It also highlights the need for targeted, context-sensitive strategies to address persistent barriers and calls for better-tailored policies to support the health and well-being of refugees in host countries and beyond.

**Supplementary Information:**

The online version contains supplementary material available at 10.1186/s12939-026-02915-x.

## Background

By mid-2024, the number of forcibly displaced persons had surpassed 120 million globally [1]. The figure has been rising at an unprecedented rate due to widespread persecution, conflict, violence, and human rights abuses. Of the 37.9 million refugees worldwide, 71% are hosted in neighboring middle- or low-income countries, where resources and capacity to support them are frequently limited [2]. Consequently, refugees are often socially and economically marginalized in host communities, living in suboptimal conditions such as impoverished urban areas or overcrowded camps with limited access to services and resources [3].

As conflicts and political and economic crises in refugees’ countries of origin become increasingly protracted, the need for achieving a sustainable long-term solution has been a primary focus for the international community. The three durable solutions are widely recognized as pathways for refugees to attain long-term stability: voluntary repatriation, local integration, and resettlement to a third country. However, with modern conflicts often becoming protracted, as seen in Venezuela and Afghanistan, voluntary repatriation, one of the durable solutions, has become less feasible. Local integration, another durable solution, may also be unattainable due to various factors, including the sheer volume of refugee populations − 3.8 million in Iran and 3.1 million in Türkiye - along with concerns about prioritizing refugees over vulnerable nationals and increased competition for limited resources [4].

The last durable solution is resettlement to a third country, helping refugees rebuild their lives and futures in the new country. Refugees who cannot seek voluntary repatriation and have specific needs that cannot be met in their country of first asylum may be eligible for resettlement to a third country (hereafter, resettlement). However, less than 1% of the global refugee population is afforded this opportunity [3]. In addition, while refugees resettled in a third country are often considered better off, they continue to face significant barriers even after resettlement. A body of literature highlights persistent disparities in healthcare access among resettled refugees. Refugees often experience unmet health needs, limited access to information and resources, language and communication barriers, financial difficulties, and systemic discrimination [5, 6].

Despite the persistent challenges and the significance of programs and policies that can mitigate the obstacles, the factors that shape healthcare access among refugees in a host country remain less explored. Prior comparative studies, such as the MIPEX Health strand, have sought to systematically benchmark migrant health policies across countries by measuring entitlements, accessibility, responsiveness, and measures to achieve change [7]. While useful for highlighting broad patterns across different countries, MIPEX does not distinguish refugees as a separate group, instead aggregating them under broader legal migrant categories, including skilled workers and students. This limits its ability to capture the specific structural barriers and lived experiences of resettled refugees. Our findings extend this work by focusing on refugee-specific access barriers and facilitators within national health systems and illustrating how policy design and implementation shape healthcare access in distinct high-income host countries.

This study employs a comparative case study approach to analyze three host countries: Australia, the United States (US), and South Korea, each with distinct histories of refugee resettlement, legislation, and policies. By examining macro- and meso-level factors in each country [8], this research seeks to understand how these determinants shape refugees’ experiences and access to healthcare in these distinct contexts, offering insights and recommendations for improving healthcare access for resettled refugee populations globally.

## Methods

We employed a comparative case study approach to identify the country-level factors that influenced healthcare access for refugees in the host countries. A case study is a research approach “used to generate an in-depth, multi-faceted understanding of a complex issue in its real-life context” [9]. A comparative case study involves a systematic comparison of two or more cases to elicit insights [10], and we followed the steps delineated in the literature [9, 10].

### Case definition and selection

The selection of host countries was based on several considerations, including immigration and integration policies concerning refugees, healthcare systems, and geographic and cultural diversity. First, including countries from different continents - Asia (South Korea), Oceania (Australia), and North America (the US) - can allow for a more comprehensive understanding of how unique geographic and socio-political contexts influence access to healthcare in each country. Additionally, each country has a distinct healthcare system, resulting in varying levels of access to healthcare for refugees. These include the largely privatized US system with public options for marginalized populations, Australia’s universal Medicare model, and South Korea’s single-payer national insurance system with supplemental private options. An in-depth analysis of these variations can help us better understand how different healthcare models affect refugees’ access to healthcare in a host country.

In this study, we define refugees as individuals who have been granted refugee status or admitted through formal third-country resettlement programs, including those admitted through resettlement pathways and those granted refugee status after arrival. We acknowledge that refugee populations are heterogeneous and that individuals under other protection regimes, such as asylum seekers or those granted temporary or humanitarian protection, experience distinct legal entitlements and access to services. For example, asylum seekers are excluded from this study because their protection status remains pending, and their legal entitlements and access to services differ substantially from those of recognized or resettled refugees.

### Data collection

To facilitate an in-depth understanding of the cases, we collected a wide range of data, including a comprehensive literature review of policies and laws, as well as peer-reviewed journal articles and interviews with stakeholders. Literature review for the Korean case has been done by SY, SK, GK and SY did a literature review for the US case. RB and AD did a literature review for Australia. A total of 47 interviews were conducted between May 2022 and October 2023 with 22 refugees and 25 stakeholders, including healthcare providers, refugee resettlement agency staff, and legal professionals (15 participants in South Korea and 32 in the United States). SY conducted the interviews done in the United States and SY, SK, and GK conducted the interviews done in South Korea. Semi-structured interview guides were developed separately for refugee women and stakeholders. The refugee interview guide included questions on demographic characteristics, migration history, adjustment experiences, experiences accessing and receiving health care, barriers and facilitators to access to healthcare, and suggestions for improving services. The stakeholder guide explored professional roles, existing refugee support programs, perceived barriers and facilitators to healthcare access, institutional and policy-level challenges, and recommendations for system-level improvements. Both guides used open-ended questions with probing to elicit detailed experiences while allowing comparability across participants and country contexts. All interview participants provided informed consent before participating. Interviews were conducted in participants’ preferred languages. Stakeholder interviews were conducted in Korean (South Korea) and English (United States). Refugee interviews were conducted in English or in participants’ native languages, depending on preference and proficiency. When needed, trained interpreters assisted during interviews. Interviews lasted approximately 30 to 80 min. The study was approved by the Institutional Review Board at the University of Arizona, Tucson, USA (Protocol number: 2104716241) and Seoul National University, Seoul, South Korea (Protocol Number: 2304/004 − 002). All interviews were recorded and transcribed with consent, and the transcripts were imported into MAXQDA 2022 (VERBI Software) for analysis. Participants were involved in testing interview questions to ensure they accurately reflected their experiences and priorities, and they also assisted with recruitment. Interview findings were disseminated with the participants in formats tailored to their literacy levels and preferences. The Australian case was not based on primary data collection conducted for this study. Instead, it draws upon the long-term qualitative research experience of AD who has led a multi-year research program with refugee communities in Australia. This prior empirical engagement informed the contextual interpretation of policies and practices discussed in this research.

### Data analysis and interpretation

The data were analyzed using the framework analysis method [11]. After transcribing interviews and preparing materials from the literature review, the data were reviewed to ensure familiarity. Coding was based on the conceptual framework developed by the first author, which informed the initial codes and themes [8]. The data were then organized, coded, and charted around the key themes identified in the framework. However, new themes identified in the data were also incorporated into the analytical framework. Interview data were used to contextualize and interpret policy and documentary findings, providing experiential and practice-based insights that informed the cross-country analysis. Given the diverse data sources utilized in this study and space limitations, this paper focuses on the common themes and country-level factors identified through the synthesis of these sources, rather than presenting standalone qualitative excerpts. Detailed analyses and findings from the qualitative data collected in the United States have been published previously [8, 12]. The interviews conducted in South Korea were conducted to provide a comparative context for this case study, and the interview guides are available in the Supplementary File.

## Results

This section examines country-level factors influencing healthcare access and social determinants of health for refugees, offering recommendations derived from the case analysis. The comparison focuses on key themes, including healthcare systems, language accessibility, social programs and structures, pathways to permanent residency and citizenship. Key country-level characteristics are summarized in Table 1.

**Table 1:** Country-level characteristics

Themes		Australia	South Korea	United States
Health care system	Facilities	• Refugee-specific facilities available through public facilities or multicultural service centers	• Limited refugee-focused facilities • Some outreach clinics exist • Big hospitals target foreign medical tourists	• Refugee-serving clinics available in some areas • Cultural navigators available in some facilities
	Providers	• General practitioners as primary contact • Referrals required for specialists	• Walk-in access is common • Referrals needed only for serious conditions	• Complex access system • Insurance-dependent provider available • Fragmented specialty care
	Educational/Research Institutions	• Cultural competency embedded in health education and professional codes of conduct	• No formal training in cultural competence • Some informal awareness training through videos	• Cultural competency guidelines (AAMC) • CLAS standards supported by HHS
	Financing Mechanisms	• Universal coverage through Medicare • Subsidized prescriptions • Optional private insurance for additional coverage	• Universal system through National Health Insurance • Access through employee-based and community-based insurance • Some refugees facing documentation and cost barriers	• No universal health coverage • Short-term refugee coverage (Refugee Medical Assistance) • Coverage gaps for certain groups of refugees
Language Access		• 24/7 free professional interpreters • Multilingual resources • Strong state-level support (e.g. NSW, Victoria policies)	• No legal framework • Informal interpretation through volunteers, family, translation apps • Limited language support in a few hospitals	• Title VI mandates language access in federally funded services • Interpretation services available but awareness remains low
Social Program for Integration		• Humanitarian Settlement Program Supports early integration (housing, language, employment) • Some long-term benefits	• Resettled refugees receive structured support (language, housing, employment) • Recognized refugees rely on NGOs • Limited long-term integration support	• Short-term integration supported by ORR (Matching Grant, cash assistance) • Focus on rapid employment, limiting long-term skill development
Transition into Permanent Residency and Citizenship		• Refugees are granted permanent residency upon arrival • Eligible for citizenship after residency period	• Difficult to obtain permanent residency due to financial/language requirements • Possibility of children remaining in undocumented status	• Eligible for permanent residency after 1 year • Eligible for citizenship after 5 years • Language/civics tests as barriers, though there are exemptions for some groups

### Healthcare system

A healthcare system can be defined as “a set of mechanisms that transform health-related resources into health services to address illness in society” [13]. The systems vary significantly between countries, and comparing these systems can provide valuable insights into different policy options, enabling policymakers to make more informed decisions. While each country’s healthcare system is distinct, they share several core components, including healthcare facilities where care is delivered, healthcare providers who provide services, educational and research institutions responsible for workforce training and knowledge advancement, and financing mechanisms that fund and sustain healthcare services [14]. In this section, we will examine how refugees access, utilize, and experience healthcare systems in three host countries, focusing on these components.

#### Health care facilities

Most of the refugees we interviewed expressed satisfaction with the healthcare facilities and services provided in the host countries [8]. They consistently perceived that the quality of health care facilities and services in host countries as better than what was available in their home countries.

Notably, the US has some health facilities specifically dedicated to serving refugees, offering tailored services to meet their unique needs. Some facilities employ “cultural navigators” - individuals from refugee communities who act as cultural brokers [15]. They help refugees navigate the healthcare system, assisting with tasks such as scheduling appointments, providing interpretation services, and connecting them with social services as needed.

In South Korea, international clinics exist in major tertiary hospitals; however, their primary purpose is not to provide services to populations with unique needs. Instead, these clinics are primarily designed to offer tailored services for affluent foreign “medical tourists.” Furthermore, the languages offered are mainly those in high demand for this purpose, such as English, Chinese, Russian, and Arabic. Additionally, since these are specialized programs for foreigners, the cost of medical services tends to be higher. Although a few hospitals offer financial assistance to individuals facing economic challenges and provide free outreach clinics for underserved populations, including immigrant workers and refugees, the number of such facilities remains limited in South Korea.

In Australia, refugee health services provide care upon arrival, and for up to five years, caseworkers deliver resettlement services to support refugees in navigating the healthcare system [16–18]. The state and territory governments provide targeted multicultural healthcare services to refugees, including initial health assessments, general medical check-ups, and access to primary healthcare services, such as general practitioners (GPs) and community-based mental health programs. These services are accessible through primary health care facilities or multicultural service centers in the local health districts.

#### Health care providers and the delivery system

In this study, health care providers are broadly defined as all entities involved in delivering health-related services. Overall, refugees expressed satisfaction with the services provided by health care providers in each country. However, there are significant variations in terms of the structures through which these services are delivered.

In the US, accessing healthcare services tends to be more complex [12]. Patients need to verify which health care providers are covered by their insurance and are accepting new patients. Accessing healthcare typically involves making a phone call to schedule an appointment, visiting health care facilities at the scheduled time, and completing extensive paperwork. When medications or extra lab work are required, patients often need to visit a pharmacy or laboratory located at a separate location, adding another layer of logistical complexity [8, 19]. For refugees with limited health literacy and language proficiency, navigating such a complex healthcare system can be both overwhelming and challenging.

South Korea’s healthcare system has simpler structures. In most cases, unless patients seek specialized services at a tertiary hospital, an appointment is not required; they can walk into a clinic. Clinics are often located in areas accessible via public transportation, and pharmacies are typically near clinics, making it easy for patients to obtain their prescriptions without traveling far. Patients can take their prescription to any nearby pharmacy and get prescribed medication. Routine blood work, tests, and labs are typically handled directly at clinics, as they are generally equipped with the necessary facilities. Only in cases where significant health issues are detected would patients be referred to higher-level care. As a result, navigating the healthcare system in South Korea is relatively straightforward and convenient for patients, requiring minimal skills.

Australia’s public healthcare system relies primarily on primary care services, which are mainly delivered by GPs. These GPs serve as the first point of contact and provide referrals for specialist care, although wait times may be prolonged. Access to hospitals and emergency services occurs at the tertiary level, while allied health, pathology, imaging, and diagnostics also require GP referrals. Appointments are needed to see a GP, and clinics are generally easy to find locally or via the government’s Healthdirect website, which offers multilingual content but may pose challenges for refugees with limited English or internet skills. Pharmacies are widely accessible in suburbs. Despite these well-established primary care services, refugees continue to face barriers, including language, health literacy, and unfamiliarity with the system, which affect their ability to navigate and benefit fully from Australia’s healthcare system [20, 21].

#### Educational and research institutions

We have examined this topic in the context of cultural competency training within educational and research institutions.

In the US, medical and nursing schools incorporate guidelines and curricula designed to equip health care providers with the skills to deliver culturally competent and appropriate care. For instance, the Association of American Medical Colleges (AAMC) published a guideline in 2005 titled “Cultural Competence Education,” which provides a clear definition of cultural competency and outlines the core attributes of a cultural competence curriculum [22]. Additionally, the U.S. Department of Health and Human Services (HHS) advocates for the National Standards for Culturally and Linguistically Appropriate Services (CLAS) in healthcare, which provide a comprehensive framework designed to guide healthcare organizations and training programs in delivering equitable, culturally responsive, and linguistically appropriate care to diverse populations [23].

In South Korea, cultural competency training is not a core component of health care provider education. Although some interviewed health care providers recalled having seen videos regarding culturally appropriate care, it is not part of official training.

In Australia, cultural competency is considered crucial for ensuring that health professionals can provide safe and respectful care to patients from diverse backgrounds. It is an integral component embedded in the curriculum and the code of conduct for all health professionals in Australia. Cultural competency is also required by all nationally accrediting bodies for allied health, nursing, midwifery, and medical practitioners from tertiary-qualified students. Furthermore, all public hospitals require training in cultural competency for all new health professionals, although there are no clear regulations governing the frequency of updates to this training [24].

#### Financing mechanisms

Costs associated with healthcare services and access to health insurance, which can mitigate financial burdens, are among the most significant determinants of health. The three countries have largely distinct health insurance systems, with different implications for the health of refugees in each country.

In the US, 8% of people are uninsured, a private plan covers 65.4%, and 36.3% are covered by a public plan, such as Medicare or Medicaid [25]. Regarding refugee health, the country has a program titled “Refugee Medical Assistance,” which provides health insurance coverage for up to eight months post-resettlement. After this period, refugees may be enrolled in Medicaid, public healthcare coverage, or purchase private insurance based on their eligibility. However, a significant number of refugees experience challenges accessing insurance. One study in the US found that 49% of refugees were uninsured, even though refugees are more likely to report chronic conditions compared to other immigrant groups [26]. Our interviews with refugees revealed that one of the groups facing the greatest challenges with health expenditure was those caught in the ‘coverage gap.’ They earned too much to qualify for Medicaid but too little to afford private insurance, which is often prohibitively expensive in the US.

South Korea does not have a health insurance scheme exclusive to refugees. Like other citizens, refugees in South Korea have access to universal healthcare through two main categories: employee-based and community-based subscribers. Individuals who are employed fall under “employee-based subscribers”. Their insurance premiums are calculated based on their monthly salary, with the cost shared between the employee and the employer. Those not covered as employee-based subscribers, such as rural farmworkers and self-employed individuals, are classified as community-based subscribers. For this group, insurance costs are determined based on factors such as income and assets. This universal healthcare system is accessible to everyone residing in South Korea, including refugees, significantly reducing the financial burden of healthcare services. However, numerous challenges persist. Many refugees work in temporary or day labor positions, which often do not qualify them for coverage under the employee-based category. Additionally, to qualify for health insurance benefits in South Korea, individuals must reside in the country for at least six months. This means that foreigners, including refugees, are only eligible for insurance after living in the country for six months. Another challenge lies in the structure of community-based health insurance. The costs are calculated per household, but refugees often face difficulties proving family relationships, such as obtaining birth certificates or marriage certificates from their home countries. As a result, they may end up paying higher insurance payments as they cannot prove that they belong to the same household. In addition, foreigners who do not hold South Korean citizenship are required to pay the average premium of all insured individuals from the previous year rather than the costs calculated based on their income or assets and end up paying a higher amount. Although some discounts are available for refugees, the financial burden remains substantial [27].

In Australia, access to health services is covered under Medicare, Australia’s universal health insurance scheme, which is available to all Australian citizens, permanent residents, and people from countries with reciprocal agreements, including refugees. In addition to free health services in the public healthcare system, Medicare also ensures access to the Pharmaceutical Benefits Scheme (PBS), which subsidizes the cost of many prescription medications. Some services, such as dental services and ambulance costs, are not subsidized through Medicare. Hence, some individuals choose to opt for private health insurance. Private health insurance also provides an option to avoid long waiting lists in the public system. As of Dec 2024, about 45.2% of the population had some form of private insurance [28]. While the universal health insurance provides comprehensive coverage for public health services and subsidized medications, refugees may still encounter indirect financial challenges such as fees for specialist consultations, transportation costs, and other out-of-pocket expenses not covered under Medicare.

### Language access

Language barriers have been reported as one of the most significant challenges that refugees often encounter when accessing healthcare in a host country. Effective communication is essential at every stage of healthcare access, from scheduling a medical appointment to communicating with health care providers. For individuals with limited language proficiency, tasks such as sharing medical history, describing symptoms, understanding different treatment options, and following up can be overwhelming and daunting.

While the language barrier is often seen as a challenge to accessing quality healthcare in the US, the country has a legal framework supporting language access for individuals with limited English proficiency (LEP). Title VI of the Civil Rights Act prohibits discrimination based on race, color, or national origin in any program or activity that receives federal funding. This act views the denial of access to federal programs for LEP individuals, based on their national origin or language proficiency, as a form of discrimination and a violation of Title VI. As a result, programs that receive federal assistance must take measures to ensure LEP individuals have meaningful access to their services [29]. Since many clinics and hospitals in the US receive some form of federal funding, they are required to offer interpretation services to LEP individuals, including refugees. However, there are clinics and hospitals, as well as refugees themselves, that are unaware of this. Beyond a few clinics that frequently see and interact with refugees, awareness is notably lower, particularly for specialty care [12].

In South Korea, no such legal frameworks exist. Interpretation is often provided informally through friends, family members, and volunteers who can speak Korean or English to assist with communication in hospitals and other healthcare settings. Additionally, free app-based services like Google Translate are commonly used. While a few hospitals that frequently serve immigrants or tourists offer on-site interpreters, the range of available languages is typically limited to only a few widely spoken ones. As a result, refugees in South Korea often struggle to express their health concerns and get adequate information from health care providers due to language barriers [27]. Furthermore, relying on male partners or family members for interpretation can sometimes lead to misunderstandings and tension, as family members may intervene and not fully or accurately convey information, especially on sensitive topics such as family planning, where spouses may have differing opinions. Overall, language barriers present substantial challenges for many refugees in navigating the healthcare system in South Korea.

Like the US, Australia has federal, state, and territory laws to support people from refugee backgrounds in accessing healthcare that is free of racism and discrimination [30, 31]. These Acts mandate that healthcare organizations provide inclusive healthcare services irrespective of race, background, or immigration status. Furthermore, the Multicultural Access and Equity Policy ensures that all Australians, regardless of their cultural and linguistic background, have access to equitable and inclusive healthcare [32]. In line with this policy, public organizations such as New South Wales Health provide 24/7 free, confidential, professional interpreting services in over 120 languages to individuals who do not speak English fluently. These services can be accessed either on-site or by telephone. Multilingual health resources are also available through regional Refugee Health Services. In Victoria, the Multicultural Health Action Plan 2023–2027 includes key initiatives such as a health translations portal and language services to ensure culturally sensitive healthcare is accessible to diverse populations, including refugees [33]. However, limited English proficiency and lower levels of health literacy continue to be significant barriers to accessing health services, as not all primary care providers utilize available translation and interpretation services due to time constraints. Also, there is a lack of awareness among refugee populations about the availability of multilingual services and other support services, such as mental health and disability support services [34].

### Social program for integration

Refugees, uprooted and disconnected from the networks and opportunities they once had in their country of origin, require substantial support to access new economic and social opportunities in a host country and become contributing members of society. In this section, we compare the social integration programs offered in the three countries, which constitute important social determinants of health for refugees.

In the US, the Office of Refugee Resettlement (ORR) supports newly arrived refugees in four key areas: health and behavioral health, employment and economic/social stability, social adjustment and integration, and services for children and youth. These efforts are carried out in collaboration with ten resettlement agencies [35]. The primary goal of these programs is to help refugees achieve self-sufficiency. Key initiatives include Refugee Cash Assistance, which provides financial support to meet basic needs, and the Matching Grant Program, which offers cash assistance, employment services, job skills training, and job referrals to facilitate economic stability and integration [36]. Despite the availability of various programs, several challenges remain. Since the primary goal of these programs is to get refugees employed as quickly as possible, there is limited time for them to develop English proficiency and acquire the skills necessary for better-paying jobs. As a result, many refugees end up in low-paying manual labor positions that often do not align with their professional experience in their home countries. Moreover, although many interviewed families had a breadwinner working full-time, their earnings were often insufficient to cover basic living expenses, including rents, as the cost of living in the US tends to be very high. Additionally, while refugees have access to exclusive short-term support, these programs are time-limited. Although refugees may still qualify for other social benefits based on household income and family size, some are unaware of these benefits or have difficulty renewing them, which requires extensive paperwork, once navigation support is no longer available [19].

In South Korea, the level of support for refugees varies significantly depending on the refugee category. While refugees who come through the resettlement program receive comprehensive assistance, including cultural orientation and Korean language training, housing support, and job placement, recognized refugees, those granted refugee status after arrival, often have access to far fewer resources. They may rely on NGOs and religious groups for support. Both groups are technically eligible for the same social benefits as Korean citizens, but many refugees are unaware of their rights, and local government officials may not fully understand that refugees are entitled to the same access as citizens. Additionally, the Korean language, as it is not widely spoken outside of Korea, presents a significant challenge for many refugees, hindering their ability to integrate and access better employment opportunities. The education provided is often insufficient, and refugees frequently struggle to pursue further training due to work commitments, and vocational training opportunities are limited [27].

In Australia, newly arrived refugees and their families are supported through the Humanitarian Settlement Program during their initial settlement. This program is implemented on behalf of the Australian government by five selected service providers across the country. The program focuses on helping eligible refugee visa holders to integrate smoothly into Australian life. The services include airport reception, short-term accommodation support, English language learning support, and assistance with accessing education, healthcare services, employment opportunities, and long-term accommodation. A longitudinal study conducted by the Australian Institute of Family Studies highlighted that the settlement experiences have been positive for many refugees, with economic participation increasing from 22% in the first year of the study (2013) to 54% in 2023 [37]. Additionally, over the ten years of settlement, more than 35% of the participants completed some form of education or job training in Australia. However, the report also highlighted that certain groups, such as women, those with limited English proficiency, minimal work experience and education on arrival, and those with poor health, were significantly less likely to be in the labor force. These findings suggest that barriers continue to exist for specific vulnerable refugee subgroups.

### Transition into permanent residency and citizenship

Access to citizenship in a host country is essential for refugees to fully integrate into their new community [38], and host countries have a responsibility to facilitate a smooth naturalization process to support this integration. However, the procedures differ across countries.

In the US, refugees can apply for lawful permanent resident status after being physically present in the country for at least one year. The fees associated with filing and fingerprinting/biometric services are waived given their financial circumstances [39]. After living in the country as a lawful permanent resident for five years, refugees become eligible to apply for citizenship. Although a common barrier to citizenship for refugees is the requirement to demonstrate English proficiency and knowledge of US history and government, exemptions are available for certain age groups and those with qualifying medical conditions [40].

Naturalization among refugees in South Korea can be particularly challenging, as they are required to demonstrate financial stability through a minimum income or assets, along with Korean proficiency. Permanent residency requires having at least 60 million won (about $46,000) in assets or a monthly income of 3 million won (approximately $2,300). This poses a significant barrier for refugees who often arrive with limited resources, as they have been forcibly displaced from their homes. Children of refugees in South Korea may end up in an undocumented status, as their parents are unable to obtain permanent residency.

In Australia, refugee category visas enable refugees to enter the country as permanent residents [41]. While there is no charge for refugee visa applications offshore, the processing time can be several years. This visa subclass is typically granted to individuals referred to the Australian government for resettlement by the UNHCR, provided they meet the health and character requirements. Once the applicants are given a refugee visa, this permanent resident visa allows them to live, work, and study in Australia without any restrictions. These visas also make the refugees eligible to enroll in the public healthcare scheme (Medicare) and propose family members for permanent residence. Once granted permanent residency, individuals may apply for Australian citizenship when they meet the eligibility criteria. These criteria include a basic proficiency in English, a good character with no serious criminal history, and a minimum of four years of residency in Australia as a permanent resident.

## Discussion

In this comparative case study, we examined three host countries to explore the country-level factors influencing refugees’ access to healthcare services. Our findings highlight that each country presented a unique mix of enablers and barriers that shape refugees’ experiences. While many refugees expressed satisfaction with the overall quality of services and facilities compared to those in their countries of origin, the availability of culturally and linguistically appropriate care varied substantially.

The US and Australia have made efforts to provide culturally and linguistically competent care through health professional training and legal mandates designed to improve access. In contrast, South Korea lacked comparable measures to ensure culturally responsive services. Health insurance emerged as another critical factor. In the US, refugees who earn too much to qualify for Medicaid but too little to afford private insurance often faced significant coverage gaps. Meanwhile, in South Korea, high premiums and challenges in providing documentation for eligibility have created barriers.

Social support programs, which are vital for helping refugees navigate their new environments and integrate into society, also varied significantly across the three countries. Refugee families in the US faced challenges related to high living costs and difficulties in renewing social benefits, while in Australia, burdens were particularly severe for the most marginalized groups, including women, individuals with limited language proficiency, and those with lower educational attainment. Overall, support structures for refugees tended to be short-term, with limited evidence of sustained impact on their long-term health and well-being.

High-income countries have increasingly adopted policies in the past 5–10 years to include refugees in national health systems and improve healthcare access. A 2022 UNHCR survey of 49 countries found that 77% include refugees in their national health plans (increased from 62% in 2019), with 94% allowing refugees to use primary care under the same conditions as citizens. However, many challenges persisted post-resettlement, as shown in this study.

Addressing these barriers requires strategies to promote equitable access to healthcare in host countries (Table 2). Key factors, such as entitlement to healthcare services, supportive laws and policies, and culturally responsive care, are instrumental in enabling refugees to navigate healthcare systems and achieve better health outcomes in host countries. For instance, Theodosopoulos et al. (2024) emphasized the role of culturally competent health care professionals in overcoming cultural and linguistic barriers faced by migrants and refugees, leading to improved health outcomes [42]. Additionally, laws and policies shape entitlements to healthcare services among refugees and their ability to access and utilize healthcare in host countries. However, as highlighted by Tomkow et al. (2019), even when legal entitlements exist, such as free emergency National Health Service care for asylum seekers and refugees in the United Kingdom, many health care professionals and refugees may be unaware of these rights. This lack of awareness exacerbates barriers to accessing care [43]. While laws and regulations are necessary, implementing appropriate measures to enhance awareness of these services can lead to improved access to care and utilization of those services.

This study synthesized an extensive body of literature, drawing also from interview data to offer broader, multi-level perspectives. Nonetheless, several limitations should be noted. While many countries host refugees, our analysis focused on a selected few, which may limit the generalizability of the findings. However, the distinct characteristics of each selected country, particularly regarding insurance systems, laws, and regulations, enabled meaningful comparisons and in-depth analysis. In addition, while the Australian section was informed by sustained qualitative research experience and long-term engagement with refugee communities, it was not based on interviews conducted specifically for this study. This may introduce differences in data sources across countries; however, we have sought to mitigate this by grounding the analysis in established research and policy evidence. In addition, this study does not include asylum seekers or individuals under other protection schemes, which limits the ability to capture the full spectrum of healthcare access across forced migrant populations. While the focus here is on recognized and resettled refugees, asylum seekers and individuals with other legal statuses often face distinct and more pronounced barriers, including more restrictive entitlements and greater uncertainty regarding their legal status. These differences may substantially shape access to care and related health outcomes. Future research should further examine how varying protection statuses influence access to healthcare and broader health determinants.

Lastly, the countries included in this study were selected to enable in-depth comparison across distinct healthcare financing systems and policy environments. However, we were not able to incorporate all major resettlement countries. For example, Canada - known for its longstanding private sponsorship model [44] and universal healthcare system - represents an important comparative case that may yield additional insights into how settlement structures and welfare regimes shape refugees’ healthcare access. Future research extending this comparative framework to include other significant host countries, such as Canada and European countries, would further strengthen the understanding of how differing policy architectures influence refugees’ navigation of the health system and outcomes.

In addition, one thing to note is that refugee policies are not static and are shaped by evolving political, legal, and social contexts within host countries. In democratic settings, shifts in political leadership and policy priorities can substantially influence refugee resettlement programs, access to services, and integration pathways. For example, changes in federal administrations in countries such as the US and Australia have historically led to variations in funding, eligibility criteria, and the scope of refugee support programs. These dynamics highlight the importance of considering the broader policy environment when interpreting cross-country differences in healthcare access and integration outcomes.

**Table 2:** Determinants of access to health and policy recommendations

Areas	Recommendations
Healthcare system	• Equip clinics to provide more tailored support to improve access to quality care. • Recognize varying levels of complexity in healthcare systems and implement additional measures to help refugees navigate them effectively (e.g., sustained training program instead of brief orientations, employing cultural navigators for ongoing navigational support). • Embed cultural competency training into the education of health care providers and institutional healthcare practices. • Pay special attention to individuals facing additional barriers or coverage gaps due to income thresholds or difficulties obtaining proper documentation, often linked to language barriers or risks of persecution.
Language Access	• Treat access to healthcare as a fundamental human right. The inability to obtain quality healthcare due to language barriers needs to be recognized as a violation of this right. • Establish robust mechanisms such as professional interpretation services to ensure all patients, including refugees, have equitable access to high-quality healthcare. • Recognize that existing laws and policies may not guarantee implementation in practice. Continuous awareness-raising efforts and advocacy should accompany policy implementation to close this gap.
Social Program for Integration	• Plan and support integration as a long-term process, fostering the sustained development and full participation of refugees in society. • Provide targeted, tailored support for vulnerable groups, including those with limited English proficiency, minimal work experience and education upon arrival, and poor health status.
Transition into Permanent Residency and Citizenship	• Recognize that transitioning to permanent residency and citizenship is crucial for complete integration. Host countries should adopt measures that actively facilitate these processes. • Mitigate foreseeable barriers wherever possible, including requirements related to proof of assets or income, and language proficiency hurdles, given the unique vulnerabilities and circumstances of refugees.

## Conclusion

Resettlement, one of the three durable solutions to severe refugee crises, offers critical support but represents only the initial step. For refugees to survive and thrive in a new environment, access equitable healthcare, and fully participate in and contribute to society, more intentional and coordinated efforts are essential. Genuine hospitality is not merely about admitting refugees, but also about ensuring they are not left behind by supporting their full integration and guaranteeing equal care and opportunities.

## Supplementary Information

Below is the link to the electronic supplementary material.

Supplementary Material 1

Supplementary Material 2

## Data Availability

This study used publicly available data for the literature review; all such sources are cited within the manuscript and can be accessed through the corresponding publications. The qualitative interview data generated and analyzed during the study are not publicly available due to confidentiality protections for participants. De-identified excerpts may be available from the corresponding author upon reasonable request and with appropriate ethical approvals.
