# Supplementary material for: Country-level determinants of healthcare access for refugee populations in a host country: a comparative case study
Source: Int J Equity Health. 2026 Jun 6;25:187. doi: 10.1186/s12939-026-02915-x (PMC13449308; doi:10.1186/s12939-026-02915-x)
Supplement: Supplementary file 1 [file 12939_2026_2915_MOESM1_ESM.docx]

**Supplementary File 1.** Summary of the major characteristics in terms of refugees in the countries

|  | **Australia** | **United States** | **South Korea** |
| --- | --- | --- | --- |
| **The number of refugees** | - 1947-2024: 950,000 | - 1980-2023: 3.2 million (resettled refugees) | - 1994-2024:   - 2,696 (granted humanitarian status)   - 122,095 (refugee applicants)   - 1,544 (recognized refugees) |
| **The year the Refugee Act was enacted** | - Migration Act, 1958 | - 1980 | - 2012 (Refugee Act was enacted and came into effect in 2013.) |
| **Health insurance coverage** | - Universal health coverage through Medicare - Subsidized pharmaceutical benefits | - No universal health care - Refugee Medical Assistance  (up to 8 months) - Medicaid for eligible groups - Private insurance - Limited access to asylum seekers | - Universal health care (payment depending on the income, and other circumstances) for recognized refugees and humanitarian status holders only - Limited access to asylum seekers (asylees can be a local subscriber) - Refugee applicants can receive financial support for the health check-up required for their asylum application. If they are not eligible for health insurance or medical assistance, they may receive financial support for hospitalization and surgery costs |
| **Eligibility for permanent residency/citizenship** | - Are granted permanent resident visa as a refugee - Eligible to live, work and study without any restrictions - Eligible to apply for citizenship after 4 years - Basic proficiency in English required - Must score 75% or more on the citizenship test | - Eligible for permanent resident one year after being admitted as a refugee (the application fees waived) - Eligible for citizenship 5 years after being granted permanent residency - Basic proficiency in English | - Eligible for permanent resident two years after being admitted as a refugee - Must also meet the following qualifications: income level (family income should be higher than per capita GNI of previous year), proficiency in Korean, be at least 19 years or older - Eligible for citizenship 5 years after living in the country - Income levels - Proficiency in Korean |
| **Entitlement depending on the status** | - Supported during initial settlement through the Humanitarian Settlement Program - Settlement payments and concessions provided (one-off crisis payment on arrival, job seeker payment, parenting support, childcare subsidy) | - Refugee Cash Assistance (up to 4 months) to meet the basic needs - Office of Refugee Resettlement (ORR) Matching Grant program (cash assistance, cash management, employment services) for self-sufficiency - Refugees are eligible for other social benefits - Enrolled initially through the refugee resettlement agencies | - Few specific supports for refugees - Resettled and recognized refugees are eligible for other social benefits - May not have the access to information and resources |
| **Refugee-serving agencies/entities** | - Federal, State and Territory governments - Non-governmental organizations (NGOs) | - Federal government (Bureau of Population, Refugees, and Migration (PRM), Office of Refugee Resettlement (ORR), United States Citizen and Immigration Services (USCIS) - State governments - NGOs (including 10 refugee resettlement agencies which get supports from the federal/state governments) | - Ministry of Justice (greater focus on adjudication of asylum rather than long-term adjustment and integration of refugees) - NGOs providing legal-aid and offering free legal services and psychosocial support (e.g., APIL, NANCEN Refugee Rights Center, GongGam), Migration to Asia Peace (MAP) - Lack of governmental support; there are often tensions with NGOs regarding refugee caps |
| **Resettlement and integration support** | - Five selected service providers appointed by the Australian government - Short-term accommodation support, assistance with accessing healthcare services, support for enrolling in education and social services, help with finding employment opportunities and long-term accommodation - Airport pick up on arrival | - Ten national resettlement agencies providing core services including:   - Initial reception and placement (airport pick up, provide temporary housing, and offer initial support with basic needs)   - Case management (assessing needs and connecting refugees with services)   - Employment services (job readiness training, job placement)   - English language training   - Healthcare (connecting with the healthcare system) | - No specific resettlement agencies available - Refugees through resettlement program can be supported through different phases: 1) pre-departure program, 2) stay at Resettlement Support Center (in Korea), 3) local resettlement (housing, job readiness training, Korean language training, etc.) - Little support for refugees other than resettled refugees |
