# Supplementary material for: Country-level determinants of healthcare access for refugee populations in a host country: a comparative case study
Source: Int J Equity Health. 2026 Jun 6;25:187. doi: 10.1186/s12939-026-02915-x (PMC13449308; doi:10.1186/s12939-026-02915-x)
Supplement: Supplementary file 2 [file 12939_2026_2915_MOESM2_ESM.docx]

# Instrument 1: Interview Guide for Refugee Women

## Research Title:

Access to and Utilization of Healthcare Services among Refugee Women: A Comparative Case Study of the United States and Korea

This interview guide is designed for use with adult refugee women participants. Questions are intended to be open-ended and non-coercive. Participants may skip any questions they do not wish to answer.

## INTRODUCTION

Thank you for considering participation in this study. The purpose of this interview is to understand the healthcare experiences of refugee women living in Korea and the United States. Your participation is voluntary. You may stop or decline to answer any question at any time.

## DEMOGRAPHIC QUESTIONS

1. What is your age?
2. What is your marital status?
3. What is your highest level of education?
4. Are you currently working?
5. Do you have health insurance? If yes, what type?
6. How many children do you have?
7. Are you currently pregnant?
8. When did you give birth to your youngest child?
9. What type of delivery did you have? (e.g., vaginal birth, cesarean section, induction)
10. How would you describe your Korean language ability? (Very good / Good / Poor / None)
11. Which country or region are you originally from?
12. How long have you lived in Korea?
13. Did you come directly, or through another country?
14. What type of visa are you currently on?

## General Experiences

1. How have you been adjusting since arriving in Korea?
2. What are your biggest health-related concerns for yourself or your family?
3. Are you currently pregnant? (If yes: How is your pregnancy progressing? How many weeks pregnant are you? If no: Have you been pregnant or given birth in Korea?)

## Knowledge and Perceptions of Prenatal Care

1. What are your thoughts on visiting a hospital during pregnancy?
2. What do you think are the benefits of prenatal care?
3. How often do you think pregnant women should see a doctor?
4. How does prenatal care differ between your home country and Korea?
5. Do women from your home country living in Korea typically receive prenatal care?
6. Are there women who do not receive prenatal care? If so, why?
7. What challenges make it difficult to receive prenatal care?
8. What would help reduce these challenges?
9. Has COVID-19 changed your experience or views regarding prenatal care?
   a. Individual-level challenges:
      i. discrimination
      ii. language barriers
      iii. cultural beliefs
   b. Environmental-level challenges:
      i. transportation
      ii. cost
   c. Institutional-level challenges:
      i. policies
      ii. service systems

## Experiences Receiving Prenatal Care

1. Did you visit a hospital during pregnancy? (If yes: When did you first go? How many times?)
2. How far was the hospital, and how did you travel there?
3. Who accompanied you? Who cared for your children?
4. Overall, how would you describe your experience?
5. Did you feel respected during your visit?
6. What challenges did you encounter?
7. Did the hospital provide culturally or linguistically appropriate information?
8. How did the hospital contact you?
9. How much were the medical expenses, and how did you pay?
10. Do you have a mobile phone? Are you able to use it to respond to hospital calls?

## Planning and Problem-Solving

1. (If pregnant) When is your next appointment and how will you get there?
2. How will you schedule future appointments? What challenges might you face?
3. (If previously pregnant) If your children or you become sick, what would you do?

## Facilitators

1. What helped you access healthcare services during pregnancy?
2. Where do you usually find information about pregnancy and childbirth?
3. Who supports you with childcare or transportation?
4. Do you receive help from family, friends, community groups, or government programs?

## Birth and Postpartum Experiences

1. Did you or your baby experience any complications?
2. What was your childbirth experience like?
3. Before discharge, what postpartum education did you receive?
4. Did you visit a hospital after giving birth?
5. What postpartum support did you receive?

## Mental Health and Family Planning

1. Have you ever been screened for depression or mental health concerns?
2. Have you learned about available contraceptive options in Korea?

## Suggestions

1. What would help improve refugee women’s access to maternal healthcare in Korea?
2. Is there anything you would like to request from the Korean government?
3. Is there anything else you would like to share?

# Instrument 2: Interview Guide for Stakeholders

## Research Title:

Access to and Utilization of Healthcare Services among Refugee Women: A Comparative Case Study of the United States and Korea

This guide is for use with individuals who work with or support refugee populations.

## INTRODUCTION

Thank you for considering participation in this interview. We are interested in your professional insights regarding refugee women’s access to healthcare. Participation is voluntary, and you may skip any question or withdraw at any time.

## DEMOGRAPHIC QUESTIONS

1. How long have you been working in refugee-related services?
2. Do you work for a private organization or a public organization?
3. What are your primary responsibilities?
4. What is your job title, if applicable?
5. What is your age?

## General Questions

1. How would you describe the overall living conditions of refugees in Korea?
2. What government programs exist to support refugees?
3. What community-level programs support refugees?

## Healthcare-Related Questions

1. What factors help refugees access healthcare?
2. Do refugees generally receive appropriate medical care when they are sick? (If not, why not?)
3. Do refugee women use maternity care appropriately? (If not, what barriers exist?)
4. What is needed to reduce these barriers?
5. What government-level or community-level support is needed?
6. How do refugees typically communicate with healthcare providers?

## Education for Refugees

1. What types of education help refugees settle and adapt quickly in Korea?
2. What information do refugees need to navigate healthcare services?

## Stakeholder Challenges

1. What challenges have you experienced while supporting refugees?
2. What support or resources would help resolve these challenges?
3. Has COVID-19 created new challenges in refugee support?

## Suggestions

1. What additional measures are needed to improve refugee health and healthcare access?
2. Is there anything else you would like to share?
